# Factors Influencing Injury Severity and Frequency among Korean Sports Participants in Their 20s and 30s

**DOI:** 10.3390/healthcare12060664

**Published:** 2024-03-15

**Authors:** Jeonga Kwon, Jusun Jang

**Affiliations:** 1 Department of Elementary Education, College of First, Korea National University of Education, Cheongju-si 28173, Republic of Korea; gilddong1234555@knue.ac.kr; 2 Department of Sports Science, Hanyang University ERICA, Ansan 15588, Republic of Korea

**Keywords:** injury frequency, injury severity, lifestyle athlete, professional athlete

## Abstract

This study aimed to explore the factors that affect the severity and number of sports injuries among Korean sports participants in their 20s and 30s. The data of 5118 lifestyle and professional athletes in their 20s and 30s were collected from a sports safety survey conducted by the Korea Sports Safety Foundation in 2019. The characteristics of the study population were analyzed using descriptive analysis. Determinants of injury severity and frequency based on participants’ sex were identified using multivariate logistic regression analyses. The results show that among male sports participants, the type of athlete, knowledge of first aids, the physical condition, completing safety education, the skill level, and checking environmental factors were associated with injury severity. Among female sports participants, the type of athlete, the physical condition, performing finishing exercises, completing safety education, and the skill level were associated with injury severity. Additionally, among male sports participants, physical condition, completing safety education, skill level, participating in exercises according to one’s fitness level, and having an awareness of accident prevention methods were associated with injury frequency. Lastly, among female sports participants, drinking water during scheduled breaks, carrying water to stay hydrated, physical condition, completing safety education, and skill level were associated with injury frequency. Furthermore, being a lifestyle athlete, being in good physical condition, and having beginner or intermediate skills in one’s sport reduced injury severity among Korean sports participants in their 20s and 30s. Being in good physical condition, not completing safety education, and having beginner skills in one’s sport reduced the injury frequency among Korean sports participants in their 20s and 30s. These findings suggest that lifestyle and professional athletes should be aware of these factors and participate in sports activities in a manner that reduces injury severity and frequency. Moreover, these factors should be considered when developing strategies to prevent sport injuries in lifestyle and professional athletes and allow them to participate in sports activities safely.

## 1. Introduction

At some point, individuals who participate in sports activities will likely experience injury. Sports injuries are physical discomforts that result from sports activities. They last for more than two weeks and can cause participants to miss subsequent matches or training sessions [1]. Most studies of football injuries have used a “time loss” definition of injury, meaning that a recordable injury is one that causes absence from football participation [2]. Sports injuries can take many forms: sprain, fracture, tear, deformity, twist, soft tissue injury, acute injury, concussion, and severe trauma [3]. These injuries can occur during individual and team sports and have significant physical, psychosocial, and financial consequences [4]. Physical disability and restricted sports activities owing to injury lead to decreased performance [5]. Moreover, sports injuries tend to cause medium- to long-term sequelae and negatively affect one’s life post-injury [6]. Notably, more than 7 out of 10 sports participants in Korea who experience an injury reduce or stop their sports activities after self-funded treatment [7]. Therefore, identifying the factors influencing sports injuries and preventing their occurrence is imperative.

Sports injuries are caused by personal, physical, psychological, and social factors, such as physical weaknesses; biomechanical imbalances; anatomical factors; failure to follow the rules; unfair opposition; previous injuries; inappropriate environments; a lack of warm-up, recovery, and protective safety equipment; and inadequate fitness level, training, skill, footwear and clothing [8]. However, a comprehensive analysis of the determinants of sports injuries reveals that they vary depending on factors such as sex, age, and sport. For instance, Yu et al. [9] suggested that the factors affecting sports injuries in combat sports athletes from Korea are a lack of warm-up, lack of flexibility, lack of physical strength, excessive desire to win, excessive tension, and high anxiety. Prieto-González et al. demonstrated that a high number of weekly practice hours increases injury rates among Spanish adolescents [10]. Not performing warm-up exercises, using inadequate sports facilities, age, having an improper training load, not performing injury prevention activities, not performing sports activities under the supervision of sports coaches, and having improper sports equipment also increase injury rates. Among South African university basketball players, the rate of injuries was greater in female basketball players than in male basketball players, and the causes of injuries were collisions with players, being hit by a basketball ball, and sudden changes in direction of rotation, which caused musculoskeletal injuries [11]. Woollings et al. showed that age, increasing years of climbing experience, highest climbing grade achieved (skill level), high climbing intensity scores, and participation in lead climbing are risk factors for injury in sports climbing and bouldering [12]. These findings suggest that sports injuries vary among subjects and are caused by a combination of factors. Furthermore, despite scientific improvements in the training methods, equipment, and instructors’ and athletes’ injury awareness, the frequency of sports injuries has not decreased. This emphasizes the need to explore the determinants of the frequency of sports injuries in detail [13].

Sports participation and injury rates are high among individuals in their 20s and 30s, and their injury experiences can have immediate and long-term negative effects [14]. As participants in these age groups are actively involved in sports, awareness of the determinants of sports injuries will enable injury prevention and better long-term results than those of adults in other age groups. Therefore, this study identified factors that influenced the severity and number of sports injuries among sports participants in their 20s and 30s in South Korea. The results of this study will serve as the foundation for policy research on preventing sports injuries and contribute to creating a healthy society.

## 2. Materials and Methods

### 2.1. Design and Data Collection

This study used data from a survey on sports safety conducted by the Korea Sports Safety Foundation in 2019. The survey was administered to lifestyle and professional athletes over the age of 13 and was conducted over three months, starting in September 2019. The interviewer met with the respondents individually and explained the questionnaire. The respondents completed a self-report paper questionnaire. At the top of the questionnaire, the foundation stated its commitment to privacy and confidentiality. The raw results and data of the survey (with personal information removed) are available on the website of the Korea Sports Safety Foundation. We downloaded these data for use in our study. Of the 11,745 individuals who participated in the survey, we extracted the data of 5118 individuals who were in their 20s and 30s. Survey data from male and female lifestyle and professional athletes in their 20s and 30s who completed all responses were included, and missing or other survey content was excluded. Because we used secondary data that did not include identifying information such as name, telephone number, home address, and social security number, ethical approval was not required. Nonetheless, this study was conducted in accordance with the principles of the Declaration of Helsinki.

### 2.2. Variables

The variables in this study were sex, type of athlete, education level, injury severity, injury frequency, performing warm-up exercises, checking facility defects, participation in exercises according to one’s fitness level, carrying water to stay hydrated, drinking water during scheduled breaks, wearing the correct clothing and safety equipment for the sport, checking environmental factors, performing finishing exercises, knowledge of first aids, awareness of accident prevention methods, awareness of how to deal with accidents, awareness of safety rules, physical condition, completing safety education, checking the safety manual, and skill level.

Injury severity was measured by the question, “How severe was your sports injury in the past one year?”. Responses regarding injury severity were based on the individual’s experience with sports injuries, which was specified to include everything from severe to very minor injuries. The response options were severe injury, moderate injury, and slight injury. Injury frequency was determined by the question, “How many sports injuries did you have in the past year?”. Respondents provided whole-number responses, which we categorized as one, two, or three or more.

Performing warm-up exercises was determined by asking, “Do you warm up before a workout?”. Checking facility defects was measured by asking, “Do you check the defects and safety of the facilities before you start exercising?”. Participation in exercises according to one’s fitness level was measured by asking, “Do you determine your fitness level before exercising and exercise accordingly?”. Carrying water to stay hydrated was determined by asking, “Before you start exercising, do you carry enough water to stay hydrated?”. Drinking water during scheduled breaks was determined by asking, “Do you drink water during scheduled breaks?”. Wearing the correct clothing and safety equipment for the sport was measured by asking, “Do you wear the correct clothing and safety equipment for your sport?”. Checking environmental factors was measured by asking, “Do you check environmental factors, such as weather and signs of disasters, before exercising?”. Performing finishing exercises was determined by asking, “Do you do finish exercises?”. Knowledge of first aid was measured by asking, “Do you know how to perform first aids, such as cardiopulmonary resuscitation?”.. The response options for these questions were not at all, rarely, sometimes, often, and always. We categorized not at all and rarely as no and sometimes, often, and always as yes.

Respondents’ awareness of accident prevention methods was determined by asking, “How much do you know about preventing sports accidents?”. The awareness of how to deal with accidents was measured by asking, “How much do you know about dealing with accidents?”. The awareness of safety rules was determined by asking, “How much do you know about the safety rules of the sport you play?”. The response options for these questions were “I do not know at all” and “I do not know a lot”, which were categorized as unaware, and “I know a little”, “I know a lot”, and “I know very much”, which were categorized as aware. 

Respondents’ physical condition was determined by asking, “How were you feeling before the injury?”. The response options were “not at all good” and “not good”, which we categorized as “poor”, and “good”, which included the responses “average”, “good” and “very good”. Whether one had completed safety education was determined by asking, “Have you received any education for sports accidents and injuries other than the regular training courses?”. The response options were “I have received such education” and “I have not received such education”. Whether one had checked the safety manual was determined by asking, “Did you check the safety manual before you became injured?”. The response options were yes and no. The skill level in sports was determined by asking, “What is the level of skill difficulty in sports?”. The response options were beginner level and pre-intermediate level, which we categorized as beginner level; intermediate level and upper intermediate level, which we categorized as intermediate level; and advanced level, which remained advanced level.

### 2.3. Data Analysis

All statistical analyses were performed using SPSS for Windows (version 23.0; IBM Corp., Armonk, NY, USA). First, a descriptive analysis was performed to determine the characteristics of the study population. Second, multivariate logistic regression analyses were conducted to identify the determinants associated with injury severity or injury frequency, based on sex. Factors associated with injuries were considered statistically significant variables as a result of the study. Statistical significance was set at *p* < 0.05.

## 3. Results

### 3.1. Participant Characteristics

Table 1 presents the characteristics of the study population. Most respondents were male (51.7%), and had a slight injury (67.8%). Respondents who experienced sports injuries three or more times in the past year were most prevalent (37.2%), followed by those who were injured once (34.3%) and twice (28.5%). Most respondents were lifestyle athletes (87.9%), and university graduates or higher (69.1%). Most respondents performed warm-up exercises (88.3%), carried water to stay hydrated (89.2%), drank water during scheduled breaks (87.2%), performed finishing exercises (67.1%), were aware of how to deal with accidents (77.8%), were aware of safety rules (67.6%), and had knowledge of first aid (84.5%). The physical condition of most respondents was good (90.4%). The percentage that had completed safety education was 49.7%, and that of those who had beginner level skills was 55.6%. Most respondents had checked the safety manual (75.6%), participated in exercises according to their own fitness level (89.2%), checked facility defects (86.9%), wore the correct clothing and safety equipment for the sport (85.6%), checked environmental factors (82.5%), and were aware of accident prevention methods (82.1%).

### 3.2. Factors Influencing Sports Injury Severity among Males

Table 2 presents the determinants of injury severity among males. The type of athlete, the physical condition, completing safety education, and the skill level were associated with severe sports injuries among males. Lifestyle athletes were 0.593 times (95% confidence interval (CI): 0.401–0.877, *p* = 0.009) more likely to have severe injuries than professional athletes. Those in good physical condition were 0.465 times (95% CI: 0.305–0.708, *p* < 0.001) more likely to have severe injuries than those in poor physical condition. Those who had beginner and intermediate skills in their sports were 0.202 times (95% CI: 0.135–0.302, *p* < 0.001) and 0.368 times (95% CI: 0.252–0.538, *p* < 0.001) more likely to have severe injuries, respectively, than those who had advanced skills. These factors can be interpreted as positively reducing the risk of severe sports injuries among males. However, those who had completed safety education were 1.728 times (95% CI: 1.286–2.323, *p* < 0.001) more likely to have severe injuries than those who had not. This factor can be interpreted as actually increasing the risk of severe sports injuries among males.

Knowledge of first aid, physical condition, completing safety education, the skill level, and checking environmental factors were associated with moderate sports injuries among males. Those who had knowledge of first aid were 0.623 times (95% CI: 0.441–0.880, *p* = 0.007) more likely to experience moderate injuries than those who did not. Those in good physical condition were 0.573 times (95% CI: 0.420–0.780, *p* < 0.001) more likely to experience moderate injuries than those in poor physical condition. Those who had beginner skills in their sport were 0.644 times (95% CI: 0.497–0.834, *p* = 0.001) more likely to experience moderate injuries than those who had advanced skills. Those who checked environmental factors were 0.767 times (95% CI: 0.592–0.993, *p* = 0.044) more likely to experience moderate injuries than those who did not. These factors can be interpreted as positively reducing the risk of moderate sports injuries among males. However, those who had completed safety education were 1.458 times (95% CI: 1.210–1.756, *p* < 0.001) more likely to experience moderate injuries than those who had not. This factor can be interpreted as actually increasing the risk of severe sports injuries among males.

### 3.3. Factors Influencing Sports Injury Severity among Females

Table 3 presents the determinants of sports injury severity among females. The type of athlete, performing finishing exercises, the physical condition, completing safety education, and the skill level were associated with severe sports injuries among females. Lifestyle athletes were 0.325 times (95% CI: 0.174–0.609, *p* < 0.001) more likely to experience severe injuries than professional athletes. Those in good physical condition were 0.363 times (95% CI: 0.223–0.592, *p* < 0.001) more likely to experience severe injuries than those in poor physical condition. Those who had completed safety education were 0.638 times (95% CI: 0.430–0.946, *p* = 0.025) more likely to experience severe injuries than those who had not. Those with beginner and intermediate skills were 0.224 times (95% CI: 0.128–0.390, *p* < 0.001) and 0.540 times (95% CI: 0.306–0.952, *p* = 0.033) more likely to experience severe injuries, respectively, than those who with advanced skills. These factors can be interpreted as positively reducing the risk of severe sports injuries among females. However, those who performed finishing exercises were 2.045 times (95% CI: 1.226–3.411, *p* = 0.006) more likely to experience severe injuries than those who did not. This factor can be interpreted as actually increasing the risk of severe sports injuries among females.

The physical condition and skill level were associated with moderate sports injuries among females. Those in good physical condition were 0.533 times (95% CI: 0.395–0.720, *p* < 0.001) more likely to experience moderate injury than those in poor physical condition. Those with beginner skills were 0.382 times (95% CI: 0.273–0.534, *p* < 0.001) more likely to experience moderate injury than those with advanced skills. These factors can be interpreted as positively reducing the risk of moderate sports injuries among females.

### 3.4. Factors Influencing Sports Injury Frequency among Males

Table 4 presents the determinants of sports injury frequency among males. Completing safety education and the skill level were associated with sports injuries that occurred twice a year among males. Those who completed safety education were 1.406 times (95% CI: 1.142–1.731, *p* = 0.001) more likely to be injured twice a year than those who had not. Those who had intermediated skills were 1.414 times (95% CI: 1.034–1.933, *p* = 0.030) more likely to be injured twice a year than those who had advanced skills. These factors can be interpreted as actually increasing the risk of severe sports injuries among females.

The physical condition, completing safety education, the skill level, participating in exercises according to their own fitness level, and having an awareness of accident prevention methods were associated with sports injuries occurring three or more times a year among males. Those in good physical condition were 0.710 times (95% CI: 0.505–0.998, *p* = 0.049) more likely to experience injuries three or more times a year than those in poor physical condition. Those who had beginner skills were 0.555 times (95% CI: 0.425–0.724, *p* < 0.001) more likely to be injured three or more times a year than those with advanced skills. Those who participated in exercise according to their fitness level were 0.603 times (95% CI: 0.395–0.921, *p* = 0.019) more likely to be injured three or more times a year than those who did not. Those who were aware of accident prevention methods were 0.657 times (95% CI: 0.438–0.987, *p* = 0.043) more likely to be injured three or more times a year than those who were not. These factors can be interpreted as positively reducing the risk of sports injury frequency among males. However, those who completed safety education were 1.989 times (95% CI: 1.640–2.412, *p* < 0.001) more likely to be injured three or more times a year than those who had not. This factor can be interpreted as actually increasing the risk of severe sports injuries among males.

### 3.5. Factors Influencing Sports Injury Frequency among Females

Table 5 presents the determinants of sports injury frequency among females. Drinking water during scheduled breaks was associated with sports injuries that occurred twice a year among females. Those who drank water during scheduled breaks were 0.730 times (95% CI: 0.544–0.980, *p* = 0.036) more likely to experience sports injuries twice a year than those who did not. These factors can be interpreted as positively reducing the risk of sports injury frequency among females.

Carrying water to stay hydrated, physical condition, completing safety education, and skill level were associated with sports injuries that occurred three or more times a year among females. Those in good condition were 0.659 times (95% CI: 0.478–0.908, *p* = 0.011) more likely to experience sports injuries three or more times a year than those in poor condition. Those who had beginner skills were 0.410 times (95% CI: 0.286–0.588, *p* < 0.001) more likely to experience sports injuries three or more times a year than those who had advanced skills. Those who had awareness of accident prevention methods were 0.643 times (95% CI: 0.465–0.888, *p* = 0.007) more likely to experience sports injuries three or more times a year than those who did not have awareness of them. These factors can be interpreted as positively reducing the risk of sports injury frequency among females. However, those who carried water to stay hydrated were 1.435 times (95% CI: 1.056–1.951, *p* = 0.021) more likely to experience sports injuries three or more times a year than those who did not. Those who completed safety education were 1.471 times (95% CI: 1.205–1.797, *p* < 0.001) more likely to experience sports injuries three or more times a year than those who had not. These factors can be interpreted as actually increasing the risk of severe sports injuries among females.

## 4. Discussion

This study aimed to explore the factors associated with the severity and number of sports injuries among Korean sports participants in their 20s and 30s. The determinants of injury severity among males were the type of athlete, knowledge of first aid, physical condition, completing safety education, skill level, and checking environmental factors. The determinants of injury severity among females were the type of athlete, physical condition, performing finishing exercises, completing safety education, and skill level. The determinants of injury frequency among males were physical condition, completing safety education, skill level, participation in exercises according to their own fitness level, and having an awareness of accident prevention methods. The determinants of injury frequency among females were drinking water during scheduled breaks, carrying water to stay hydrated, physical condition, completing safety education, and skill level. Furthermore, the type of athlete, being in good condition, and having beginner or intermediate skills were associated with the severity of sports injuries among sports participants in their 20s and 30s. Meanwhile, being in good condition, not completing safety education, and not having beginner skills were associated with the frequency of injuries among sports participants in their 20s and 30s.

First, we found that the type of athlete was associated with the severity of injuries among sports participants. In particular, the severity of injuries was lower among lifestyle athletes than among professional athletes. This finding indicates that professional athletes experience more severe injuries than lifestyle athletes. Engaging in sports activities is beneficial for one’s physical health, but a high risk of injury is involved [15]. Lifestyle athletes engage in sports activities in their leisure time. However, professional athletes engage in them daily and perform more exercises with a higher intensity. Consequently, the possibility of professional athletes getting injured is higher [9,10]. Sports injuries affect athletes’ daily lives, training, performance, physical health, mental health, and medical expenses [16,17,18]. Injuries to elite athletes have negative consequences for their team and the country they represent [19,20]. Sports injuries can lead to poor performance and early retirement, and can also dampen the spirit of spectators [21]. Suffering a sports injury affects not only one’s life at that moment, but also one’s future, by causing medium- and long-term sequelae [6]. Moreover, injuries affect one’s physical health long after they occur [16]. Von Porat et al. [22] found that the prevalence of knee osteoarthritis among 219 male footballers 14 years after anterior cruciate ligament (ACL) injury is 41% in the injured knee and 4% in the uninjured knee. Similarly, Marshall et al. [23] found that college athletes who suffered ankle injuries exhibited a lower health-related quality of life in their later life. Experiencing an injury can also cause increased anxiety, post-traumatic stress disorder, and fear of participating in sports, leading to poor performance, delayed return, and dropout [17,24,25]. McCullough et al. [24] found that 50% of athletes who undergo ACL reconstruction due to injury do not return to sports because of fears of reinjury. Notably, the prevalence of post-traumatic stress disorder among elite athletes is higher than that among the general population, as athletes who experience injury are more likely to develop post-traumatic stress disorder [26]. Injuries have a high probability of recurrence and increase the physical and mental risks faced by athletes. Thus, it becomes essential to determine and analyze the causes of injuries that can occur during training and competitions, and minimize these causes. This will be an important step toward increasing athletes’ well-being, improving performance, and preventing injuries [27].

Second, we found that, compared with being in poor physical condition, being in good physical condition was associated with a reduction in the severity and frequency of injuries among sports participants, indicating that poor physical condition may increase injury severity and frequency. Poor health in a match can instill a lack of confidence and motivation and cause excessive tension and anxiety, which can result in injury [28,29,30]. Regardless of how good a player is, being in poor health while participating in a game is likely to lead to injury. For instance, Park Joo-ho, a South Korean soccer player, played multiple games between 2018 and 2019 with an improperly treated shin stress fracture. His unfit physical condition hampered his performance and led to another injury [31]. One study found that players in South Jordan have medium burnout levels, and experiencing burnout is related to the number of sports injuries [32]. Another study found that participating in sports activities while sleep-deprived increases the risk of injury [33]. These cases suggest that athletes who engage in sports activities with poor physical or mental health are more likely to be injured, which can lead to poor performance. In particular, females who participate in sports need to be cautious when they are not feeling well due to hormonal changes during their menstrual cycle, and the risk of injury increases owing to muscle relaxation, as well as strength, body temperature, and neuromuscular regulation changes [34,35]. This is because females have a greater risk of musculoskeletal injuries, such as patellofemoral pain syndrome and ACL injury, during menstruation [36]. In addition, female athletes complain of fatigue when menstruating, which reduces their confidence and concentration in competition, leading to injuries and negatively affecting their performance [37,38]. Therefore, female professional athletes should avoid high-intensity exercises as much as possible during menstruation to minimize the risk of injury. If they are concerned about injury, not participating in sports activities is the best way to reduce the possibility of injury. Studies suggest that, if an athlete’s skill set or physical strength is inadequate, controlling psychological factors and maintaining one’s health can improve performance and reduce the likelihood of injury [39]. One study found that professional basketball players who have a positive psychological state exhibit better performance due to immersion when participating in the game [40]. Having confidence in one’s ability, a willingness to have fun, and a relaxed state of mind maximize immersion in the game. Another study found that ACL injury prevention training for female players may help reduce injury rates by improving their physical condition [41]. Therefore, players must maintain good physical and mental health to minimize the possibility of injury. This can be accomplished by participating in injury prevention programs; engaging in physical training, imagery training, and positive thinking; following routines; getting adequate rest and sleep; receiving massages; and controlling mood swings [33,42,43]. Generally, when an athlete suffers an injury, they ignore it or hide it, and do not allow recovery. However, they must take time to heal and recover [44]. Engaging in sports activities without letting the injury heal is likely to cause additional injuries. Therefore, providing athletes with opportunities to regain and maintain their health is necessary.

Third, we found that having beginner or intermediate skills was associated with reducing the severity and frequency of injuries among sports participants. This finding suggests that having advanced skills increases the injury severity and frequency. Lee et al. [45] partially agreed with these findings in their survey of ball sports players, reporting that excessive skill and movement attempts have the highest response rate. Other studies have shown that intense training and competitive activities aimed at improving the skills of professional athletes increase the likelihood of sports injuries [46,47]. Currently, athletes’ skills are growing, and scientific and technological advancements have allowed athletes to perform many difficult techniques. However, to develop such skills, athletes must practice difficult techniques despite the high risks associated with them, and in the process, they experience excessive pain [48]. For example, the Kolman technique, a complex and dangerous movement in artistic gymnastics competitions, often causes injuries; however, the technique must be performed to enter the top ranks and is performed by many athletes [49]. However, injuries caused by such techniques are one of the factors that cause young elite athletes to drop out; therefore, caution is required [50]. Rogers et al. [51] emphasized that practicing more difficult skills carries a high risk of injury, and increasing confidence and reducing fear are necessary. The use of virtual reality is a good method to improve technical performance with a reduced likelihood of injury. In fact, in many sports, including racket sports, martial arts, ball sports, and target sports, practicing skills using virtual reality has helped participants to safely improve their performance and reduced the likelihood of injury [52]. This suggests that performing difficult techniques with a low risk of injury is possible. Therefore, professional athletes must participate in sports activities appropriate to their fitness level, and they must use safer methods to practice difficult techniques to reduce the risk of injury.

Fourth, we found that not completing safety education was associated with a reduction in the frequency of injuries among sports participants. Cusimano et al. [53] used educational videos to provide concussion-related safety training to minor league hockey players; training immediately impacted the players’ concussion-related knowledge but resulted in no significant difference in the training’s effectiveness after two months. However, several studies have shown that receiving safety education effectively reduces the likelihood of injury [54]. Thus, the results of this study differ from those of previous studies, and further research is required on this topic.

This study has several limitations. First, the study population comprised lifestyle and professional athletes, but we could not identify injury determinants based on the type of athlete. Therefore, future studies should stratify their populations according to athlete type so that determinants of injury severity and injury frequency can be separately identified. Future studies should also try to identify injury determinants based on the specific sport being considered, to prevent injuries and prolong the career of athletes. Second, the measurement of the severity and number of injuries was subjective. Memory errors may have occurred while thinking about injuries suffered in the past year. Third, we had no information about the sports practiced by the athletes in this study. Thus, more well-designed studies are necessary. Despite these limitations, this study is valuable because it comprehensively analyzed and identified factors associated with injury severity and the number of injuries. The results of this study will help sports participants in their 20s and 30s to recognize injury determinants and safely enjoy sports activities, which, in turn, will contribute to building a healthy nation.

## 5. Conclusions

In conclusion, our findings suggest that lifestyle and professional athletes should be aware of the factors identified in the study and participate in sports activities in a manner that reduces injury severity and frequency. In addition, these factors should be considered when developing strategies to prevent sport injuries in lifestyle and professional athletes, and allow them to participate in sports activities safely.

## Figures and Tables

**Table 1:** Characteristics of the study population (*n* = 5118).

Variable		Frequency (%)
Sex	Male	2646 (51.7%)
	Female	2472 (48.3%)
Injury severity	Severe injury	382 (7.5%)
	Moderate injury	1264 (24.7%)
	Slight injury	3472 (67.8%)
Injury frequency	Once	1755 (34.3%)
	Twice	1461 (28.5%)
	Three or more times	1902 (37.2%)
Type of athlete	Lifestyle athlete	4499 (87.9%)
	Professional athlete	619 (12.1%)
Education level	High school graduate or less	518 (10.1%)
	Attending university	1063 (20.8%)
	University graduate or higher	3537 (69.1%)
Performed warm-up exercises	Yes	4521 (88.3%)
	No	597 (11.7%)
Carried water to stay hydrated	Yes	4565 (89.2%)
	No	553 (10.8%)
Drank water during scheduled breaks	Yes	4465 (87.2%)
	No	653 (12.8%)
Performed finishing exercises	Yes	3436 (67.1%)
	No	1682 (32.9%)
Awareness of how to deal with accidents	Aware	3982 (77.8%)
	Unaware	1136 (22.2%)
Awareness of safety rules	Aware	3460 (67.6%)
	Unaware	1658 (32.4%)
Knowledge of first aids	Yes	4326 (84.5%)
	No	792 (15.5%)
Physical condition	Good	4628 (90.4%)
	Poor	490 (9.6%)
Completed safety education	Yes	2544 (49.7%)
	No	2574 (50.3%)
Skill level	Beginner level	2847 (55.6%)
	Intermediate level	1275 (24.9%)
	Advanced level	996 (19.5%)
Checked the safety manual	Yes	1248 (24.4%)
	No	3870 (75.6%)
Participated in exercises according to their own fitness level	Yes	4564 (89.2%)
	No	554 (10.8%)
Checked facility defects	Yes	4449 (86.9%)
	No	669 (13.1%)
Wore the correct clothing and safety equipment for the sport	Yes	4382 (85.6%)
	No	736 (14.4%)
Checked environmental factors	Yes	4222 (82.5%)
	No	896 (17.5%)
Awareness of accident prevention methods	Aware	4203 (82.1%)
	Unaware	915 (17.9%)

**Table 2:** Factors influencing the sports injury severity among males.

Variable		Severe Injury		Moderate Injury	
		Odds Ratio (95% Confidence Interval)	*p*-Value	Odds Ratio (95% Confidence Interval)	*p*-Value
Type of athlete	Lifestyle athlete	0.593 (0.401–0.877)	0.009 **	0.846 (0.629–1.139)	0.271
	Professional athlete	Reference		Reference	
Education level	High school graduate or less	0.745 (0.447–1.242)	0.259	1.019 (0.762–1.363)	0.900
	Attending university	1.005 (0.714–1.415)	0.979	0.927 (0.735–1.169)	0.521
	University graduate or higher	Reference		Reference	
Performed warm-up exercises	Yes	1.701 (0.768–3.766)	0.190	1.272 (0.826–1.958)	0.275
	No	Reference		Reference	
Carried water to stay hydrated	Yes	0.736 (0.372–1.454)	0.377	1.173 (0.766–1.796)	0.463
	No	Reference		Reference	
Drank water during scheduled breaks	Yes	1.600 (0.791–3.235)	0.191	0.973 (0.671–1.410)	0.883
	No	Reference		Reference	
Performed finishing exercises	Yes	1.071 (0.719–1.595)	0.734	1.188 (0.938–1.505)	0.154
	No	Reference		Reference	
Awareness of how to deal with accidents	Aware	0.925 (0.503–1.703)	0.803	0.951 (0.661–1.370)	0.788
	Unaware	Reference		Reference	
Awareness of safety rules	Aware	0.883 (0.605–1.290)	0.521	0.962 (0.762–1.215)	0.748
	Unaware	Reference		Reference	
Knowledge of first aid	Yes	0.991 (0.549–1.789)	0.976	0.623 (0.441–0.880)	0.007 **
	No	Reference		Reference	
Physical condition	Good	0.465 (0.305–0.708)	<0.001 ***	0.573 (0.420–0.780)	<0.001 ***
	Poor	Reference		Reference	
Completed safety education	Yes	1.728 (1.286–2.323)	<0.001 ***	1.458 (1.210–1.756)	<0.001 ***
	No	Reference		Reference	
Skills level	Beginner level	0.202 (0.135–0.302)	<0.001 ***	0.644 (0.497–0.834)	0.001 **
	Intermediate level	0.368 (0.252–0.538)	<0.001 ***	0.791 (0.608–1.031)	0.083
	Advanced level	Reference		Reference	
Checked the safety manual	Yes	1.070 (0.772–1.484)	0.685	1.114 (0.899–1.380)	0.323
	No	Reference		Reference	
Participation in exercises according to one’s fitness level	Yes	0.647 (0.333–1.258)	0.200	0.982 (0.647–1.491)	0.931
	No	Reference		Reference	
Checked facility defects	Yes	0.717 (0.474–1.083)	0.114	1.042 (0.781–1.389)	0.782
	No	Reference		Reference	
Wore the correct clothing and safety equipment for the sport	Yes	1.478 (0.881–2.480)	0.139	1.017 (0.758–1.364)	0.912
	No	Reference		Reference	
Checked environmental factors	Yes	1.044 (0.682–1.598)	0.842	0.767 (0.592–0.993)	0.044 *
	No	Reference		Reference	
Awareness of accident prevention methods	Aware	0.854 (0.449–1.626)	0.632	1.141 (0.769–1.693)	0.512
	Unaware	Reference		Reference	

* *p* < 0.05, ** *p* < 0.01, *** *p* < 0.001; assessed through multivariate logistic regression analysis.

**Table 3:** Factors influencing sports injury severity among females.

Variable		Severe Injury		Moderate Injury	
		Odds Ratio (95% Confidence Interval)	*p*-Value	Odds Ratio (95% Confidence Interval)	*p*-Value
Type of athlete	Lifestyle athlete	0.325 (0.174–0.609)	<0.001 ***	0.916 (0.574–1.462)	0.713
	Professional athlete	Reference		Reference	
Education level	High school graduate or less	0.826 (0.447–1.527)	0.542	0.766 (0.532–1.103)	0.152
	Attending university	0.850 (0.502–1.442)	0.548	1.073 (0.820–1.404)	0.608
	University graduate or higher	Reference		Reference	
Performed warm-up exercises	Yes	0.641 (0.319–1.286)	0.210	0.886 (0.643–1.220)	0.458
	No	Reference		Reference	
Carried water to stay hydrated	Yes	0.900 (0.487–1.660)	0.735	1.174 (0.857–1.609)	0.318
	No	Reference		Reference	
Drank water during scheduled breaks	Yes	0.624 (0.352–1.107)	0.107	0.858 (0.639–1.152)	0.309
	No	Reference		Reference	
Performed finishing exercises	Yes	2.045 (1.226–3.411)	0.006 **	1.110 (0.883–1.396)	0.372
	No	Reference		Reference	
Awareness of how to deal with accidents	Aware	1.481 (0.764–2.871)	0.245	1.078 (0.791–1.469)	0.635
	Unaware	Reference		Reference	
Awareness of safety rules	Aware	0.989 (0.620–1.578)	0.965	0.902 (0.715–1.139)	0.387
	Unaware	Reference		Reference	
Knowledge of first aids	Yes	1.072 (0.646–1.778)	0.789	1.245 (0.964–1.607)	0.093
	No	Reference		Reference	
Physical condition	Good	0.363 (0.223–0.592)	<0.001 ***	0.533 (0.395–0.720)	<0.001 ***
	Poor	Reference		Reference	
Completed safety education	Yes	0.638 (0.430–0.946)	0.025 *	1.110 (0.906–1.359)	0.315
	No	Reference		Reference	
Skills level	Beginner level	0.224 (0.128–0.390)	<0.001 ***	0.382 (0.273–0.534)	<0.001 ***
	Intermediate level	0.540 (0.306–0.952)	0.033 *	0.807 (0.567–1.150)	0.235
	Advanced level	Reference		Reference	
Checked the safety manual	Yes	1.460 (0.944–2.258)	0.089	1.201 (0.947–1.522)	0.131
	No	Reference		Reference	
Participation in exercises according to one’s fitness level	Yes	1.440 (0.656–3.161)	0.363	0.851 (0.613–1.179)	0.332
	No	Reference		Reference	
Checked facility defects	Yes	0.937 (0.519–1.690)	0.828	1.142 (0.835–1.560)	0.406
	No	Reference		Reference	
Wore the correct clothing and safety equipment for the sport	Yes	0.740 (0.405–1.351)	0.327	0.984 (0.724–1.338)	0.919
	No	Reference		Reference	
Checked environmental factors	Yes	0.979 (0.584–1.641)	0.936	0.972 (0.750–1.260)	0.832
	No	Reference		Reference	
Awareness of accident prevention methods	Aware	1.061 (0.524–2.151)	0.869	0.976 (0.700–1.361)	0.886
	Unaware	Reference		Reference	

* *p* < 0.05, ** *p* < 0.01, *** *p* < 0.001; assessed through multivariate logistic regression analysis.

**Table 4:** Factors influencing sports injury frequency among males.

Variable		Two Times		Three or More Times	
		Odds Ratio (95% Confidence Interval)	*p*-Value	Odds Ratio (95% Confidence Interval)	*p*-Value
Type of athlete	Lifestyle athlete	1.046 (0.737–1.484)	0.802	0.968 (0.711–1.318)	0.834
	Professional athlete	Reference		Reference	
Education level	High school graduate or less	1.002 (0.724–1.386)	0.991	1.010 (0.747–1.366)	0.948
	Attending university	1.244 (0.961–1.612)	0.097	1.221 (0.960–1.552)	0.105
	University graduate or higher	Reference		Reference	
Performed warm-up exercises	Yes	0.941 (0.606–1.461)	0.787	1.406 (0.909–2.177)	0.126
	No	Reference		Reference	
Carried water to stay hydrated	Yes	0.760 (0.495–1.168)	0.211	1.344 (0.861–2.098)	0.194
	No	Reference		Reference	
Drank water during scheduled breaks	Yes	0.985 (0.660–1.470)	0.943	1.101 (0.747–1.623)	0.628
	No	Reference		Reference	
Performed finishing exercises	Yes	0.970 (0.748–1.257)	0.817	0.804 (0.631–1.023)	0.076
	No	Reference		Reference	
Awareness of how to deal with accidents	Aware	0.804 (0.546–1.185)	0.270	1.309 (0.891–1.923)	0.170
	Unaware	Reference		Reference	
Awareness of safety rules	Aware	1.110 (0.863–1.428)	0.416	1.263 (0.994–1.604)	0.056
	Unaware	Reference		Reference	
Knowledge of first aid	Yes	0.683 (0.465–1.006)	0.053	0.947 (0.641–1.397)	0.783
	No	Reference		Reference	
Physical condition	Good	0.770 (0.534–1.111)	0.162	0.710 (0.505–0.998)	0.049 *
	Poor	Reference		Reference	
Completed safety education	Yes	1.406 (1.142–1.731)	0.001 **	1.989 (1.640–2.412)	<0.001 ***
	No	Reference		Reference	
Skills level	Beginner level	0.961 (0.713–1.296)	0.796	0.555 (0.425–0.724)	<0.001 ***
	Intermediate level	1.414 (1.034–1.933)	0.030 *	0.987 (0.747–1.303)	0.924
	Advanced level	Reference		Reference	
Checked the safety manual	Yes	1.057 (0.831–1.344)	0.653	0.824 (0.657–1.033)	0.093
	No	Reference		Reference	
Participation in exercises according to one’s fitness level	Yes	1.015 (0.643–1.601)	0.949	0.603 (0.395–0.921)	0.019 *
	No	Reference		Reference	
Checked facility defects	Yes	0.883 (0.646–1.206)	0.435	0.978 (0.729–1.312)	0.882
	No	Reference		Reference	
Wore the correct clothing and safety equipment for the sport	Yes	1.128 (0.819–1.553)	0.461	1.089 (0.806–1.472)	0.577
	No	Reference		Reference	
Checked environmental factors	Yes	1.333 (0.990–1.795)	0.059	1.066 (0.814–1.396)	0.642
	No	Reference		Reference	
Awareness of accident prevention methods	Aware	0.994 (0.653–1.514)	0.979	0.657 (0.438–0.987)	0.043 *
	Unaware	Reference		Reference	

* *p* < 0.05, ** *p* < 0.01, *** *p* < 0.001; assessed through multivariate logistic regression analysis.

**Table 5:** Factors influencing sports injury frequency among females.

Variable		Two Times		Three or More Times	
		Odds Ratio (95% Confidence Interval)	*p*-Value	Odds Ratio (95% Confidence Interval)	*p*-Value
Type of athlete	Lifestyle athlete	1.421 (0.798–2.532)	0.233	1.049 (0.651–1.690)	0.844
	Professional athlete	Reference		Reference	
Education level	High school graduate or less	1.094 (0.755–1.585)	0.635	1.244 (0.876–1.768)	0.222
	Attending university	0.831 (0.623–1.107)	0.205	0.946 (0.722–1.240)	0.687
	University graduate or higher	Reference		Reference	
Performed warm-up exercises	Yes	1.051 (0.769–1.436)	0.755	1.123 (0.817–1.543)	0.475
	No	Reference		Reference	
Carried water to stay hydrated	Yes	1.317 (0.971–1.786)	0.076	1.435 (1.056–1.951)	0.021 *
	No	Reference		Reference	
Drank water during scheduled breaks	Yes	0.730 (0.544–0.980)	0.036 *	0.847 (0.629–1.140)	0.273
	No	Reference		Reference	
Performed finishing exercises	Yes	1.214 (0.969–1.522)	0.092	1.231 (0.984–1.539)	0.069
	No	Reference		Reference	
Awareness of how to deal with accidents	Aware	1.156 (0.858–1.556)	0.340	1.236 (0.914–1.671)	0.169
	Unaware	Reference		Reference	
Awareness of safety rules	Aware	1.258 (0.999–1.585)	0.051	1.226 (0.975–1.541)	0.081
	Unaware	Reference		Reference	
Knowledge of first aid	Yes	0.944 (0.741–1.203)	0.641	1.189 (0.930–1.521)	0.168
	No	Reference		Reference	
Physical condition	Good	0.880 (0.621–1.247)	0.472	0.659 (0.478–0.908)	0.011 *
	Poor	Reference		Reference	
Completed safety education	Yes	1.221 (0.996–1.498)	0.055	1.471 (1.205–1.797)	<0.001 ***
	No	Reference		Reference	
Skills level	Beginner level	0.977 (0.644–1.482)	0.913	0.410 (0.286–0.588)	<0.001 ***
	Intermediate level	0.982 (0.624–1.545)	0.936	0.812 (0.552–1.195)	0.290
	Advanced level	Reference		Reference	
Checked the safety manual	Yes	0.861 (0.671–1.106)	0.242	0.961 (0.757–1.221)	0.744
	No	Reference		Reference	
Participation in exercises according to one’s fitness level	Yes	0.962 (0.692–1.338)	0.818	0.845 (0.609–1.173)	0.314
	No	Reference		Reference	
Checked facility defects	Yes	0.940 (0.689–1.283)	0.697	0.844 (0.626–1.139)	0.267
	No	Reference		Reference	
Wore the correct clothing and safety equipment for the sport	Yes	0.836 (0.620–1.127)	0.240	0.885 (0.655–1.198)	0.430
	No	Reference		Reference	
Checked environmental factors	Yes	0.959 (0.739–1.245)	0.754	0.924 (0.715–1.194)	0.546
	No	Reference		Reference	
Awareness of accident prevention methods	Aware	0.854 (0.618–1.180)	0.339	0.643 (0.465–0.888)	0.007 **
	Unaware	Reference		Reference	

* *p* < 0.05, ** *p* < 0.01, *** *p* < 0.001; assessed through multivariate logistic regression analysis.

## Data Availability

The data that support the findings of this study are available from the corresponding author, upon reasonable request.

## References

[1] Doral M.N., Tandoğan R.N., Mann G., Verdonk R. (2011). Sports Injuries: Prevention, Diagnosis, Treatment and Rehabilitation.

[2] Hägglund M., Waldén M., Bahr R., Ekstrand J. (2005). Methods for epidemiological study of injuries to professional football players: Developing the UEFA model. Br. J. Sports Med..

[3] Abernethy L., Bleakley C. (2007). Strategies to prevent injury in adolescent sport: A systematic review. Br. J. Sports Med..

[4] Van Eetvelde H., Mendonça L.D., Ley C., Seil R., Tischer T. (2021). Machine learning methods in sport injury prediction and prevention: A systematic review. J. Exp. Orthop..

[5] Lee S.H. (2008). Sports injury and rehabilitation. J. Coach. Dev..

[6] Wiese-Bjornstal D.M., Wood K.N., Kronzer J.R., Tenenbaum G., Eklund R.C., Boiangin N. (2020). Sport injuries and psychological sequelae. Handbook of Sport Psychology: Social Perspectives, Cognition, and Applications.

[7] Lee Y.U. (2017). Sports Injury Insurance: Issues and Methods of Promotion. J. Sports Entertain. Law.

[8] Bird S.R., Black N., Newton P. (1997). Sports Injuries: Causes, Diagnosis, Treatment and Prevention.

[9] Yu J.I., Seo T.B., Cho Y.H. (2019). Study on the frequency of sports injury and re-injury in combat sports athletes. J. Korean Alliance Martial Arts.

[10] Prieto-González P., Martínez-Castillo J.L., Fernández-Galván L.M., Casado A., Soporki S., Sánchez-Infante J. (2021). Epidemiology of sports-related injuries and associated risk factors in adolescent athletes: An injury surveillance. Int. J. Environ. Res. Public Health.

[11] Ellapen T.J., Narsigan S., Essack F.M., Jugroop P., Macrae N.A., Milne J., Stow C., Van Heerden H.J. (2012). Prevalence of basketball-related musculoskeletal injuries among university players: Biokinetics practice and sport injuries. Afr. J. Phys. Health Educ. Recreat. Danc..

[12] Woollings K.Y., McKay C.D., Emery C.A. (2015). Risk factors for injury in sport climbing and bouldering: A systematic review of the literature. Br. J. Sports Med..

[13] Junge A., Engebretsen L., Alonso J.M., Renstrom P.A., Marshall S.W., Golightly Y.M. (2007). Sports Injury and arthritis. NC Med. J..

[14] Emery C.A., Pasanen K. (2019). Current trends in sport injury prevention. Best Pract. Res. Clin. Rheumatol..

[15] Kay M.C., Register-Mihalik J.K., Gray A.D., Djoko A., Dompier T.P., Kerr Z.Y. (2017). The epidemiology of severe injuries sustained by National Collegiate Athletic Association student-athletes, 2009–2010 through 2014–2015. J. Athl. Train..

[16] Toft A.M.H., Møller H., Laursen B. (2010). The years after an injury: Long-term consequences of injury on self-rated health. J. Trauma Acute Care Surg..

[17] Gledhill A., Forsdyke D. (2021). The Psychology of Sports Injury: From Risk to Retirement.

[18] Hansen M.G., Ross A.G., Meyer T., Knold C., Meyers I., Peek K. (2023). Incidence, characteristics and cost of head, neck and dental injuries in non-professional football (soccer) using 3 years of sports injury insurance data. Dent. Traumatol..

[19] Hoffman D.T., Dwyer D.B., Bowe S.J., Clifton P., Gastin P.B. (2020). Is injury associated with team performance in elite Australian football? 20 years of player injury and team performance data that include measures of individual player value. Br. J. Sports Med..

[20] Lambert C., Ritzmann R., Akoto R., Lambert M., Pfeiffer T., Wolfarth B., Lachmann D., Shafizadeh S. (2022). Epidemiology of injuries in Olympic sports. Int. J. Sports Med..

[21] Soligard T., Myklebust G., Steffen K., Holme I., Silvers H., Bizzini M., Junge A., Dvorak J., Bahr R., Andersen T.E. (2008). Comprehensive warm-up programme to prevent injuries in young female footballers: Cluster randomised controlled trial. BMJ.

[22] Von Porat A.R.E.M., Roos E.M., Roos H. (2004). High prevalence of osteoarthritis 14 years after an anterior cruciate ligament tear in male soccer players: A study of radiographic and patient relevant outcomes. Ann. Rheum. Dis..

[23] Marshall A.N., Valier A.R.S., Yanda A., Lam K.C. (2020). The impact of a previous ankle injury on current health-related quality of life in college athletes. J. Sport Rehabil..

[24] McCullough K.A., Phelps K.D., Spindler K.P., Matava M.J., Dunn W.R., Parker R.D., Reinke E.K., MOON Group (2012). Return to high school- and college-level football after anterior cruciate ligament reconstruction: A Multicenter Orthopaedic Outcomes Network (MOON) cohort study. Am. J. Sports Med..

[25] Maffulli N., Longo U.G., Gougoulias N., Loppini M., Denaro V. (2010). Long-term health outcomes of youth sports injuries. Br. J. Sports Med..

[26] Lynch J.H. (2021). Posttraumatic stress disorder in elite athletes. Curr. Sports Med. Rep..

[27] Stephenson S.D., Kocan J.W., Vinod A.V., Kluczynski M.A., Bisson L.J. (2021). A comprehensive summary of systematic reviews on sports injury prevention strategies. Orthop. J. Sports Med..

[28] Andrade A., Silva R.B., Dominski F.H. (2020). Application of sport psychology in mixed martial arts: A systematic review. Kinesiology.

[29] Ivarsson A., Johnson U., Andersen M.B., Tranaeus U., Stenling A., Lindwall M. (2017). Psychosocial factors and sport injuries: Meta-analyses for prediction and prevention. Sports Med..

[30] Kim D.H., Yun Y.K. (2020). Long jumpers performance disturbance in training and competition. Sports Sci..

[31] Lee S.J. (2021). Analysis of the Status of Injuries in Professional Soccer Players from 2011 to 2019. Korea J. Sports Sci..

[32] Alhajaya M., Al-Zghailat M. (2016). The Level of Burnout and its Relationship to Sports Injuries among Players Altaikuangtsu in South of Jordan. Dirasat Educ. Sci..

[33] Copenhaver E.A., Diamond A.B. (2017). The value of sleep on athletic performance, injury, and recovery in the young athlete. Pediatr. Ann..

[34] Raj R.D., Fontalis A., Grandhi T.S., Kim W.J., Gabr A., Haddad F.S. (2023). The impact of the menstrual cycle on orthopaedic sports injuries in female athletes. Bone Jt. J..

[35] Martínez-Fortuny N., Alonso-Calvete A., Da Cuña-Carrera I., Abalo-Núñez R. (2023). Menstrual cycle and sport injuries: A systematic review. Int. J. Environ. Res. Public Health.

[36] Thein-Nissenbaum J.M., Rauh M.J., Carr K.E., Loud K.J., McGuine T.A. (2012). Menstrual irregularity and musculoskeletal injury in female high school athletes. J. Athl. Train..

[37] Read P., Mehta R., Rosenbloom C., Jobson E., Okholm Kryger K. (2022). Elite female football players’ perception of the impact of their menstrual cycle stages on their football performance. A semi-structured interview-based study. Sci. Med. Footb..

[38] Sánchez M., Rodríguez-Fernández A., Bosque V.D., Bermejo-Martín L., Sánchez-Sánchez J., Ramírez-Campillo R., Villa-Vicente J. (2022). Effects of the menstrual phase on the performance and well-being of female youth soccer players. Cult. Cienc. Deporte.

[39] Xuey I.Y., Kwak H.P. (2019). The Influence of the Psychological Disturbances on Athletic Competitions and its Countermeasures for Chinese Professional Sports Aerobics Athlete. Korean J. Converg. Sci..

[40] Yang H.S., Li F., Feng H. (2021). Exploring the Flow Experience of Professional Basketball Players. Korean J. Sport Psychol..

[41] Myer G.D., Sugimoto D., Thomas S., Hewett T.E. (2013). The influence of age on the effectiveness of neuromuscular training to reduce anterior cruciate ligament injury in female athletes: A meta-analysis. Am. J. Sports Med..

[42] Lim S.J., Chun B.K. (2009). The Overcome Strategy on Psychological and Physical Inhibitors for Female Professional Bowlers. Korean Soc. Sport Psychol..

[43] Guo G., Xie S., Cai F., Zhou X., Xu J., Wu B., Guanghui W., Ran X., Xiruo X. (2021). PingEffectiveness and safety of massage for athletic injuries: A protocol for systematic review and meta-analysis. Medicine.

[44] Kim H.B., Kwon S.Y. (2013). Exploring the meanings of injury experiences among college soccer players. Korean J. Sociol. Sport.

[45] Lee O., Park S.Y., Lim J.J. (2022). A Study of Occurrence and Cause of Injury During Match and Training for Ball Sports Players. Korean J. Phys. Educ..

[46] Lee J.S., Kim S.S. (2014). A Study on Exercise Addiction Propensity and Exercise Injury in Martial-art Trainees. Korean J. Sport.

[47] Weiss K.J., Allen S.V., McGuigan M.R., Whatman C.S. (2017). The relationship between training load and injury in men’s professional basketball. Int. J. Sports Physiol. Perform..

[48] Ahn S.D., Lee N.K., Jun H.P. (2021). A Retrospective Study on Injury Epidemiology of Youth Taekwondo Poomsae Athletes in Gyeongsang Province. Taekwondo J. Kukkiwon.

[49] Song J.H., Park J.C. (2011). Analysis of Kinematic Factors for the Improvement of Performance Ability of Kolman Technique in the Horizontal Bars. Korean J. Sport Sci..

[50] Smyth E.A., Newman P., Waddington G., Weissensteiner J.R., Drew M.K. (2019). Injury prevention strategies specific to pre-elite athletes competing in Olympic and professional sports—A systematic review. J. Sci. Med. Sport.

[51] Rogers M., Paskevich D. (2021). Experience and Management of Fear in Men’s World Cup Alpine Ski Racing. Front. Psychol..

[52] Richlan F., Weiß M., Kastner P., Braid J. (2023). Virtual training, real effects: A narrative review on sports performance enhancement through interventions in virtual reality. Front. Psychol..

[53] Cusimano M.D., Chipman M., Donnelly P., Hutchison M.G. (2014). Effectiveness of an educational video on concussion knowledge in minor league hockey players: A cluster randomised controlled trial. Br. J. Sports Med..

[54] Provvidenza C., Tator C.H., Tator C., Tator C. (2008). Sports Injury Prevention: General Principles. Catastrophic Injuries in Sport and Recreation: Causes and Prevention–A Canadian Study.

